# A Long-Term Experimental Case Study of the Ecological Effectiveness and Cost Effectiveness of Invasive Plant Management in Achieving Conservation Goals: Bitou Bush Control in Booderee National Park in Eastern Australia

**DOI:** 10.1371/journal.pone.0128482

**Published:** 2015-06-03

**Authors:** David B. Lindenmayer, Jeff Wood, Christopher MacGregor, Yvonne M. Buckley, Nicholas Dexter, Martin Fortescue, Richard J. Hobbs, Jane A. Catford

**Affiliations:** 1 Fenner School of Environment and Society, The Australian National University, Canberra, Australian Capital Territory, Australia; 2 ARC Centre of Excellence for Environmental Decisions, The Australian National University, Canberra, Australian Capital Territory, Australia; 3 National Environmental Research Program, The Australian National University, Canberra, Australian Capital Territory, Australia; 4 Long-term Ecological Research Network, Terrestrial Ecosystem Research Network, The Australian National University, Canberra, Australian Capital Territory, Australia; 5 ARC Centre of Excellence for Environmental Decisions, University of Queensland, Brisbane, Queensland, Australia; 6 School of Natural Sciences & Trinity Centre for Biodiversity Research, Zoology, Trinity College, University of Dublin, Dublin, Ireland; 7 Parks Australia, Department of the Environment, Jervis Bay Village, Jervis Bay Territory, Australia; 8 School of Plant Biology, University of Western Australia, Crawley, Western Australia, Australia; 9 ARC Centre of Excellence for Environmental Decisions, University of Western Australia, Crawley, Western Australia, Australia; 10 School of BioSciences, The University of Melbourne, Melbourne, Victoria, Australia; 11 Department of Ecology, Evolution and Behavior, University of Minnesota, St Paul, Minnesota, United States of America; Fudan University, CHINA

## Abstract

Invasive plant management is often justified in terms of conservation goals, yet progress is rarely assessed against these broader goals, instead focussing on short-term reductions of the invader as a measure of success. Key questions commonly remain unanswered including whether invader removal reverses invader impacts and whether management itself has negative ecosystem impacts. We addressed these knowledge gaps using a seven year experimental investigation of Bitou Bush, *Chrysanthemoides monilifera* subsp. *rotundata*. Our case study took advantage of the realities of applied management interventions for Bitou Bush to assess whether it is a driver or passenger of environmental change, and quantified conservation benefits relative to management costs of different treatment regimes. Among treatments examined, spraying with herbicide followed by burning and subsequent re-spraying (spray-fire-spray) proved the most effective for reducing the number of individuals and cover of Bitou Bush. Other treatment regimes (e.g. fire followed by spraying, or two fires in succession) were less effective or even exacerbated Bitou Bush invasion. The spray-fire-spray regime did not increase susceptibility of treated areas to re-invasion by Bitou Bush or other exotic species. This regime significantly reduced plant species richness and cover, but these effects were short-lived. The spray-fire-spray regime was the most cost-effective approach to controlling a highly invasive species and facilitating restoration of native plant species richness to levels characteristic of uninvaded sites. We provide a decision tree to guide management, where recommended actions depend on the outcome of post-treatment monitoring and performance against objectives. Critical to success is avoiding partial treatments and treatment sequences that may exacerbate invasive species impacts. We also show the value of taking advantage of unplanned events, such as wildfires, to achieve management objectives at reduced cost.

## Introduction

Biological invasions are considered to be a major threat to biodiversity [1]. Consequently, invasive species management is often justified through its contribution to biodiversity conservation goals. However, there are frequently complex issues associated with invasive species control, including potentially deleterious impacts on native species [2] and ecosystems, such as herbicide effects on pollinators [3]. Invasive species control also may increase resource availability (e.g. light, water, nutrients) and can make a community more susceptible to re-invasion by the target invader or other invasive species [4].

Under the Driver-Passenger model for invasive species [5], if the invader is a passenger of environmental change (i.e. native species decline and exotic species invasion both result from environmental change that occurs independently of invasion), removal of the invader will not lead to ecosystem recovery. In such cases, it will likely be more effective to invest in an alternative management approach that addresses the underlying ecological change (e.g. altered disturbance regimes, habitat fragmentation or dispersal limitation of native species) rather than species-specific control of the invader. Conversely, if the invader is a driver of ecosystem change where invasion does not follow, but causes, ecological modification (e.g. suppression of native species whether directly or indirectly), then its removal can lead to ecosystem recovery (e.g. [6,7]), assuming that ecological change is reversible and off-target effects of invader control are minimal [5]. In rare cases where species alter environmental conditions in their favour [8], species can be both drivers and passengers of environmental change: initial invasion leads to environmental change, which facilitates more invasion, establishing a positive feedback between invasion and environmental modification. As a consequence, transformer species, and the ecosystems they invade, are particularly difficult to manage [9].

**Fig 1:**
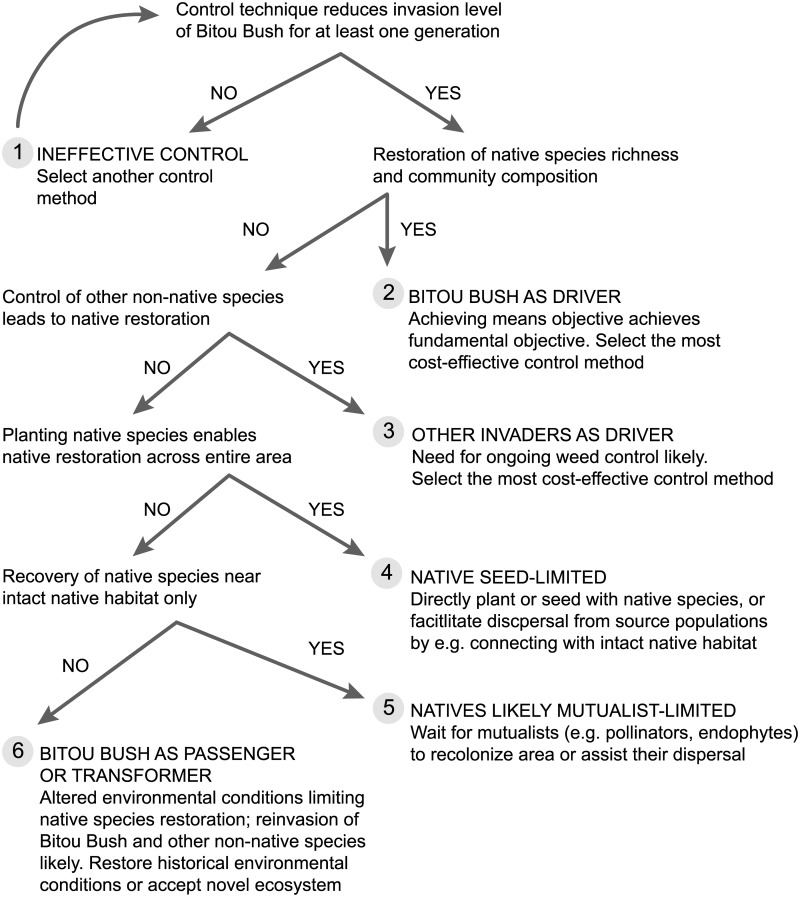
Appropriate management actions following incursion of an invasive species (Bitou Bush) and concurrent loss of native species. The decision tree can be applied to any situation where an invasive species is suspected of causing native species loss, but where understanding of the process is limited. Actions are based on post-treatment observations, but can be tailored depending on knowledge of a system (e.g. the sequence of tests for scenarios 3–5 can be altered). The decision tree accounts for the main factors that may limit recovery of native species (changed biotic conditions (competition from invaders, loss of mutualists), propagule availability and environmental suitability) and tests these in a sequence likely to be most cost-effective for native species restoration. Costs include resources and off-target impacts. Where Bitou Bush acts as a driver of ecological change (Scenarios 2–5) and its propagules continue to be dispersed into the area, ongoing control will be required. Scenarios 3–5 may be the result of prolonged Bitou Bush invasion (Bitou Bush as a driver, albeit not immediately detectable), off-target impacts of the control method or they may be independent of Bitou Bush invasion (although evidence indicates that this is unlikely); additional means objectives (see text) will be required to enable native species restoration. In Scenario 6, means objectives will need to be changed from control of the invader to management of the environment to meet the fundamental objective of management. Because inferences are based solely on post-treatment observations, it is not possible to distinguish between an invader that is a passenger or transformer with this decision tree (environmental change may be dependent or independent of invasion). However, this lack of certainty does not affect the recommended application and sequence of management actions when this framework is first applied to a case study, though it will affect future recommendations for that case study (i.e. if invaders are passengers, not transformers, only the environment need to be managed).

Despite considerable understanding of invasive species ecology, control efforts are rarely evaluated in terms of progress towards key management objectives or relative costs and benefits [10,11]. Key inter-related questions often remain unanswered, including: **(1)** whether the target invasive species has been effectively controlled, **(2)** whether a target species is a driver, passenger or transformer of environmental change and hence whether its control is likely to lead to ecosystem recovery, **(3)** whether the methods used to control invasive species have positive or negative impacts on other plants or animals (including other undesirable species), and **(4)** if invasive species management is cost-effective in terms of its benefits relative to levels of expenditure [12]. Answers to these questions have major implications for guiding management actions for invasive species (Fig 1).

**Fig 2:**
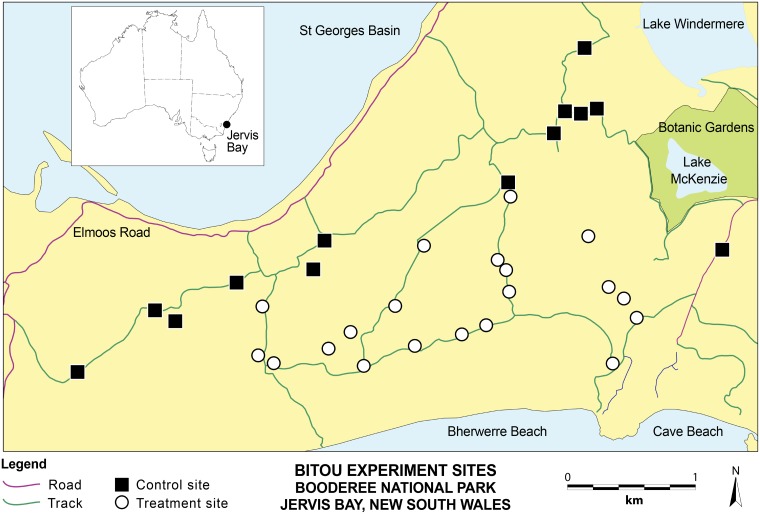
The Bherwerre Peninsula in Booderee National Park showing the location of permanent field plots in the experimental study of Bitou Bush control. The control sites are those areas without Bitou Bush. The treatment sites are those where all or part of a treatment sequence was applied (see text).

We addressed some of these major knowledge gaps using a 7-year experimental case study focused on examining aspects of the effectiveness of the Bitou Bush, *Chrysanthemoides monilifera* subsp. *rotundata* (DC) T. Norl., control program within Booderee National Park (BNP) in Australia (Fig 2). Bitou Bush is regarded as one of the 32 worst weeds on the Australian continent [13] and listed as a Weed of National Significance [14]. Despite considerable advances in understanding the causes and consequences of its invasion [15–17], experimental studies of the effectiveness of control efforts have rarely been evaluated in the scientific literature (although see [18] for several case studies), and particularly the efficacy of different sub-components of the sequence of treatments employed in an attempt to control Bitou Bush (but see [13]). In addition, the cost-effectiveness of different sub-components of the sequence of treatments has not been determined.

The fundamental objective (i.e. primary, underlying motivation) of Bitou Bush control in BNP is to restore native plant species richness to levels characteristic of uninvaded sites [19]. This aim is pursued through attempts to limit the spread of the species and confine it to low levels of abundance within those places where it currently exists. Whether these means objectives (secondary aims that are collectively designed to meet the fundamental objective, i.e. means to an end) are appropriate for achieving the fundamental objective will depend upon whether Bitou Bush is a driver, passenger or transformer of environmental change (Fig 1). If Bitou Bush is a driver of ecological change resulting in native species loss, its control (which may need to be ongoing if Bitou Bush propagules continue to arrive) should lead to ecosystem recovery, assuming minimal off-target impacts of the control method, an absence of tipping points and alternative stable states, a lack of other invasive, driver species, and availability of native seeds and mutualists (Fig 1). If Bitou Bush is a passenger of environmental change, control (ongoing or not) that targets the species will not lead to native species recovery and ecosystems will likely be invaded by other exotic plant species where exotic seed sources are available. This may also occur if the weed is a transformer of the environment such that sites are no longer suitable for the native plant species [8]. If Bitou Bush is a passenger of environmental change, a different means objective will be required to achieve the fundamental objective as Bitou Bush control will not help to restore native plant species richness. To date, it has not been possible to assess whether efforts to control Bitou Bush in BNP are meeting any of the fundamental or means objectives and therefore if program costs are warranted relative to benefits obtained.

Given that Bitou Bush was extensively planted in parts of BNP, and the reserve supports few other non-native plant species [20], our first hypothesis was that it was a driver of environmental change, which is consistent with other research (e.g. [6,7,18,21]). Therefore, if the abundance of Bitou Bush was reduced through management, we postulated that the abundance of native plant species would increase. However, off-target impacts of control or a lack of native seeds and mutualists could mask ecosystem recovery following Bitou Bush removal. Notwithstanding these limitations, testing this hypothesis would allow us to quantify the benefits of Bitou Bush control relative to its costs and also determine whether control efforts were successful in terms of meeting the means objectives and the fundamental management objectives of the control program. To test this hypothesis, we addressed four questions articulated in our general model (Fig 1).

### Question 1: Do control efforts result in a reduction in the cover of Bitou Bush and the abundance of live Bitou Bush plants?

The primary treatment for Bitou Bush at BNP comprises a series of sub-treatments including herbicide spraying, burning, and repeat spraying. For logistical and other reasons, not all of these are necessarily completed, nor is the recommended sequence of sub-treatments always conducted. A subsequent question therefore was: **Is there a difference in the response of Bitou Bush when the full sequence of sub-treatments is employed versus when only part of the treatment protocol is applied?**

### Question 2: Do control efforts promote re-invasion by Bitou Bush or other exotic plant species?

Attempts to control invasive plants can increase the susceptibility of an ecosystem to re-invasion by the same or another non-native plant species, termed the “weed-shaped hole effect” [22]. We quantified the response of Bitou Bush and other exotic plants to management treatments to determine whether the overall level of invasion (i.e. total abundance of all exotic species) rebounded post-control, which would be evidence of the “weed-shaped hole” effect (Fig 1). As other non-native species are largely absent from those parts of BNP infested by Bitou Bush [20], we predicted that if a “weed-shaped hole” were created by management, then Bitou Bush would reinvade the managed plots leading to no change in Bitou Bush abundance through time. An alternative possibility was that disturbance by the spray-fire-spray regime may be larger than the individual sub-treatments alone, potentially leading to open niches or lack of competition from native plant species.

### Question 3: What are the effects of the Bitou Bush control program on native plants?

Part of the control effort for Bitou Bush entails herbicide treatments which may have unintended negative impacts on non-target native species [21]. Consistent with earlier studies, we predicted the Bitou Bush control program would have transient negative impacts on native plant species, but net positive effects on the recovery of native plant species in the longer term [16].

### Question 4: What is the cost-effectiveness of Bitou Bush control?

The different sub-components of the treatment protocol for Bitou Bush have different costs [11,23] and potentially different impacts on Bitou Bush and native plant taxa. This enabled us to integrate data on the ecological effectiveness and the cost-effectiveness of the Bitou Bush control program.

Studies like the one we report here are important to better quantify: (1) the efficacy of invasive plant control (2) impacts of such control programs on non-target native plant taxa, and (3) cost-effectiveness relative to the ability to meet the objectives of control programs [11,23]. The case study which features in this investigation also highlights the value of collaboration between on-the-ground management by resource managers in an iconic National Park and applied long-term experimental research by scientists to tackle practical problems of conservation significance.

## Methods

### Ethics statement

No specific permits were required for our field studies as they were observational investigations of the results of the experimental management treatments of bitou bush being conducted by Parks Australia, which manages Booderee National Park. The studies were conducted on public land in Booderee National Park in collaboration with Parks Australia staff.

### Study area

Our study area was the 1000 ha Bherwerre Peninsula in BNP, 200 km south of Sydney, south-eastern Australia (midpoint is 35°10'S, 150° 40'E) (Fig 2). The region has a temperate climate with an average rainfall of approximately 1250 mm per annum spread relatively evenly over the year. Average minimum and maximum air temperatures for February (summer) are 18-24°C and for July (winter) 9.2-15°C [20].

We focused our case study on areas of Swamp Oak, *Casuarina glauca* Sieber ex Spreng., woodland and forest dominated by Bangalay (*Eucalyptus botryoides* Sm.). These areas of vegetation are characterised by shallow soils derived from Pleistocene (< 1.6 million year old) windblown sand dune systems, which cover Permian (~260 million year old) sandstone sequences [20].

Much of the Bherwerre Peninsula has been subject to grazing by domestic livestock and vegetation clearing prior to the area being gazetted as a National Park [20]. However, other kinds of disturbances such as sand mining operations have not occurred on the Bherwerre Peninsula, nor in other parts of BNP.

### Study species

**Fig 3:**
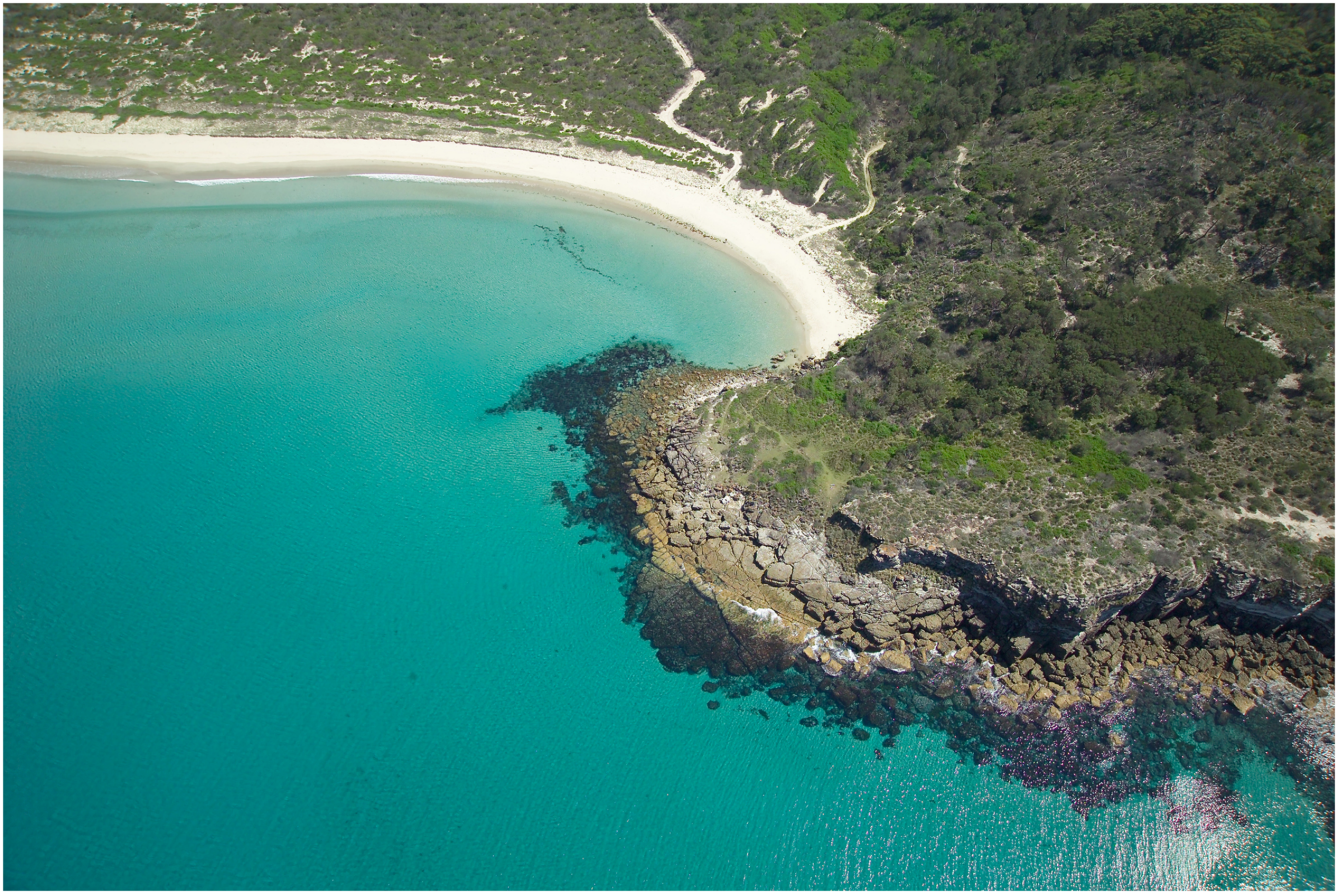
Lines of Bitou Bush (emerald green) deliberately planted to stabilise the foredunes on Bherwerre Peninsula and Beach in BNP. The planting occurred long before Bitou Bush was declared an environmental weed in Australia. (Photo from BNP Archive).

Bitou Bush is an exotic perennial shrub planted in Australia from 1946 to 1968 to stabilise coastal sand dunes (including at BNP; see Fig 3). The species is now widespread throughout coastal eastern Australia [13,14]. Its seeds are spread by animals and motor vehicles. Extensive areas of Bitou Bush were deliberately planted on the tops of dunes on the Bherwerre Peninsula [20], although never behind the dunes where we located our sites (see below).

Bitou Bush establishment can alter microclimatic conditions and rates of key ecosystem processes like litter decomposition and nutrient cycling [24], exclude native plants [21], and influence invertebrate and bird populations [25]. Bitou Bush can produce a seed bank of up to 3000 seeds m^-2^ [26]. However, seed longevity is estimated to be < 1 year [27].

### Treatment regime for Bitou Bush

The full treatment regime for Bitou Bush control in BNP is a combination of sequential sub-treatments. These sub-treatments are applied by staff from BNP, except for aerial spraying which is conducted by a professional contractor using a helicopter. A key sub-treatment is spraying of Ultra Low Volume (ULV) glyphosate by helicopter using a concentration of 15% glyphosate. Spraying takes place in winter when native plants are relatively inactive but Bitou Bush remains metabolically active [28].

**Fig 4:**
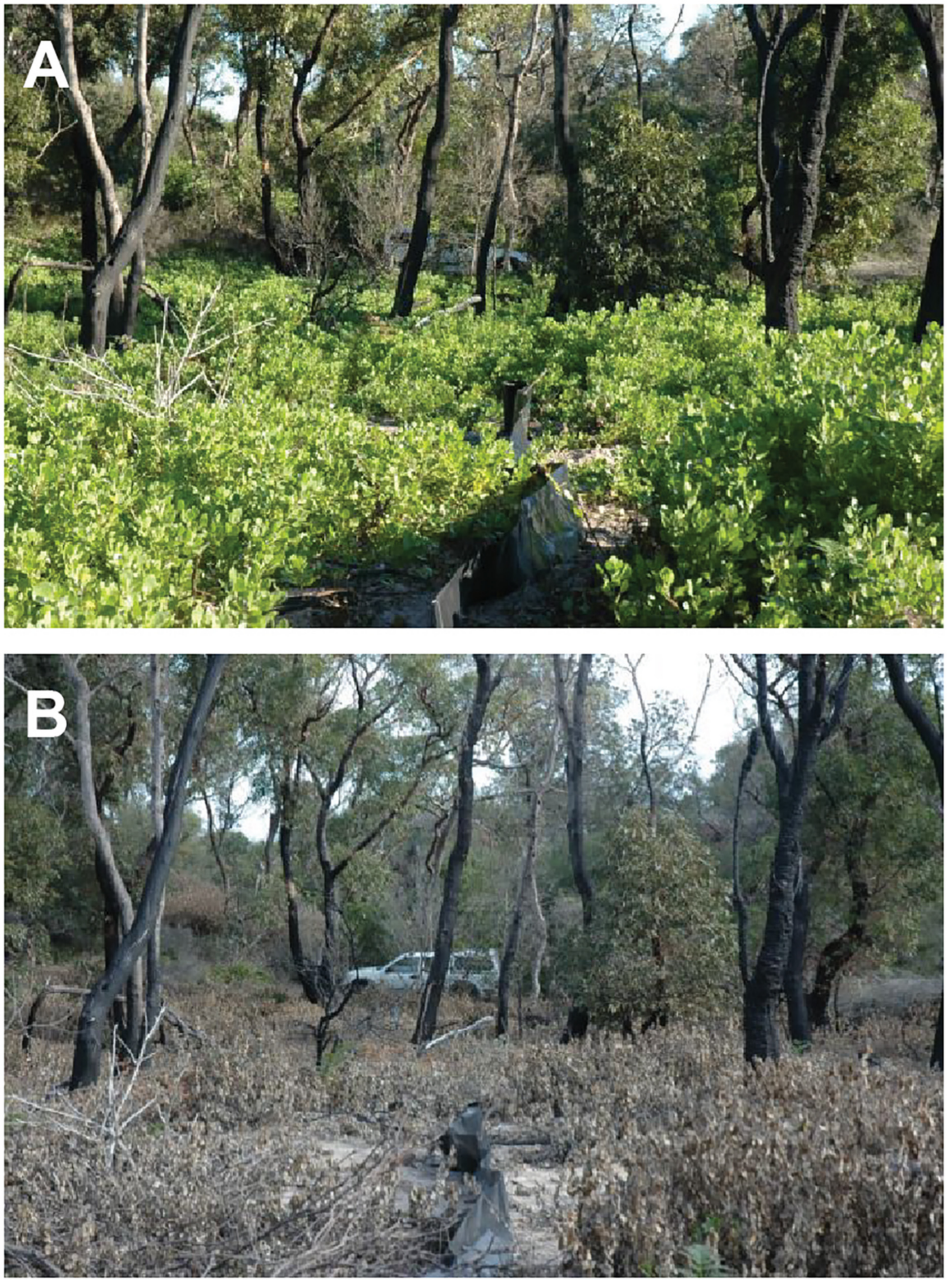
**(A) A site heavily infested by resprouting Bitou Bush following fire; (B) The same site following a spraying treatment to control Bitou Bush** (photos by C. MacGregor).

After the first application of herbicide, dead Bitou Bush plants dry for >1 year before being burned in a prescribed fire. The fire triggers germination of Bitou Bush seed in the soil and a year later, a follow-up spray of ULV glyphosate kills fire-triggered seedlings (Fig 4). Treatment burns are applied to 15–85 ha areas of vegetation where Bitou Bush has been sprayed in any given year. These areas are currently not subject to additional prescribed burning programs for fire hazard reduction or the protection of human infrastructure. Unlike many other invasive plant species control programs, there is no replanting or seeding of native plants in BNP as it is assumed that native vegetation will recover by seeds dispersing from untreated to treated areas.

### Experimental design

We established an experiment to estimate the effects of different treatment sequences on Bitou Bush control and on native plants. Working with a Weed of National Significance (see [14]) in a National Park presents some key practical and logistical constraints for the design of a large-scale field experiment. We briefly describe these five constraints and how we addressed them below.

First, under the management plan for BNP, areas invaded by Bitou Bush **must** be subject to some kind of treatment (even if the recommended protocol cannot be fully implemented; see below). This precluded the establishment of permanent control sites where Bitou Bush occurred but remained untreated. However each site was surveyed on a number of occasions at different stages of the treatment sequence, so comparisons of no treatment, a partial sequence of treatments, and a full sequence can be made from observations at the same site. We also included external control sites where Bitou Bush did not occur. Half of these non-infested sites were sprayed with ULV glyphosate. These sites were used to help assess the effect of the treatments on native vegetation in the absence of Bitou Bush. Importantly, the control sites had a similar past disturbance history (of grazing and clearing) to the treatment sites so they could be appropriately compared. All sites were located in the same broad area (with similar climatic conditions) and in the same two target vegetation types where infestation is a particular problem—Swamp Oak and forest dominated by Bangalay.

Second, the large area of Bitou Bush infestation on Bherwerre Peninsula meant that it was not possible to spray all affected areas at the same time. Even if this was possible, there were insufficient resources to subsequently burn all treated areas simultaneously.

Third, logistical issues meant that only some areas received the complete spray-fire-spray regime and others were subject to only parts of it or different combinations of particular treatments. Therefore, the sites in our experiment had a variety of different treatment histories (Table 1).

**Table 1:** Levels of replication for each of the treatment sequences used to control Bitou Bush at BNP.

Survey															
	07a	07b	08a	08b	08c	08d	09a	09b	09c	10a	10b	11a	12a	14a	Total
Disturbance sequence															
FFS	0	0	0	0	0	0	0	2	2	2	1	0	0	0	7
FSF	0	0	2	2	2	2	2	2	0	0	0	0	0	0	12
FSS	0	0	0	0	0	0	0	0	0	1	1	0	0	0	2
SFF	0	1	2	2	2	2	2	0	0	0	0	0	0	0	11
SFS	0	0	0	0	0	0	0	1	1	0	3	2	2	0	9
SSF	1	1	2	2	2	2	2	3	3	2	0	0	0	0	20
SSS	0	0	0	0	2	0	2	0	0	3	5	7	6	2	27
XFS	0	0	0	0	0	0	0	2	1	1	2	1	1	0	8
XSF	4	1	2	2	2	1	2	3	5	3	2	1	1	0	29
XSS	2	0	0	0	0	1	1	1	4	1	1	0	1	5	17
XXF	0	0	0	0	0	0	0	0	0	3	2	1	1	0	7
XXS	3	2	10	10	9	6	10	8	4	6	5	4	4	5	86
XXX	7	0	2	2	1	0	2	1	0	1	1	7	7	11	42
XXSc	0	0	0	0	0	0	0	0	0	0	6	6	6	6	24
XXXc	4	1	4	4	4	0	4	4	4	10	4	4	4	4	55
**Total**	**21**	**6**	**24**	**24**	**24**	**14**	**27**	**27**	**24**	**33**	**33**	**33**	**33**	**33**	**356**

The code for survey shows the year and the identity of the sample period in that year; for example, the second vegetation survey completed in 2007 is denoted 07b. The codes for disturbance sequence are S for spray, and F for fire. Where there were fewer than three such sub-treatments in the four years prior to a survey, we use the letter X to show the missing treatment. Thus, the sequence spray-fire-spray was denoted as SFS, but where only a single spray event was conducted the code is XXS, and in the absence of any control measures (and prior to the application of treatments), the code is XXX. For the control sites, which were not part of the Bitou Bush infested area, we added ‘c’ to the sequence code. Since half these sites were not treated and half received one spray, the only sequences observed were XXXc and XXSc.

Fourth, the recommended spray-fire-spray treatment regime requires several years to be fully implemented. This meant that because a given site is surveyed many times throughout the duration of the study, it could appear under different treatment sequences according to the progression of particular treatments over time.

Fifth, it was not possible to prevent some kinds of other disturbances and in late 2007, a moderate severity wildfire resulted in some sites being burned once by prescribed fire (post-Bitou Bush spraying) and once by wildfire.

Our experiment comprised 33 sites, each with an 80-metre long transect and encompassed three site types: **(1)** Control sites with no Bitou Bush. **(2)** Sites with Bitou Bush where the complete control regime of spray-fire-spray was applied. And, **(3)** Sites with Bitou Bush where only part of the treatment regime was applied. Each site was surveyed on up to 14 occasions, giving 356 site-survey combinations in total (Table 1). Our study focused on documenting the history of treatments at a given site in the preceding four years. Spraying was denoted S, Fire was denoted F. For example, the treatment sequence spray-fire-spray at a site is shown as SFS, and spray-spray-fire is SSF. The absence of a component of the treatment sequence was denoted X; for instance a site subject to spray and a fire was presented as SFX. Sites where there was no fire or spraying were represented as XXX. Control sites without Bitou Bush were denoted XXSc if they had been sprayed and XXXc otherwise (see Table 1).

Our sites were located in the same broad area (Fig 2) and experienced the same climatic and other conditions. However, because of the previous dune stabilisation program, sites in our experiment that did not support Bitou Bush tended to be ~500 m further from the coast than sites characterised by major infestations. Sites were typically 200 m apart to ensure spatial independence and to minimise the risk that fire or spraying inadvertently affected nearby sites.

At the beginning of our study, there was heterogeneity in the starting state of individual field sites (see column 1 in Table 1), and appropriate statistical methods were used to accommodate these site history differences by fitting random effects for both site and survey year to account for between-site differences not related to the treatments (see section below on statistical methods).

### Vegetation surveys

We established four 1 m x 1 m permanent survey plots on an 80 m transect on each of our 33 sites. Plots on each transect were set at least 20 m apart. At each plot, we placed a 1m x 1m frame on or over the vegetation. We then completed % cover measurements for live Bitou Bush, dead Bitou Bush, all native vegetation, crown cover (by observation above the plot to an area of approximately 10 m x 10 m). In addition, we completed counts of the number of individual live Bitou Bush plants, dead Bitou Bush plants, other exotic plant species, and native plant species. We derived a list of species in each 1m x 1m survey plot, from which we generated data for the number of exotic and native plant species on the transect. A single observer (CM) completed all vegetation surveys between 2007 and 2014.

We targeted two distinct vegetation types. Major levels of plant species diversity are typically associated with the ground layer in the Swamp Oak woodland and forest ecosystems and this layer was the focus of our experiment. Given this, we elected to pool data from the series of plots at each site to assess the number of individuals of each species. Our aim was **not** to create a comprehensive list of all plant species nor a full documentation of total species richness. Rather, we sampled in a consistent manner across all plots within the 33 sites in our experiment and then compared various measures of plant response through time and between sites subject to different treatment sequences.

### Cost data

We generated cost estimates (in AUD ha^-1^ in 2012) for the different sub-treatments employed in Bitou Bush control from actual costs of spraying and burning incurred according to budget records of resource managers based in BNP who applied the treatment programs. The cost of aerial spraying was $A145 ha^-1^. Fire treatments entailed burning of 15–85 ha compartments after herbicide spraying. Fire treatment costs were estimated to be $A1150 ha^-1^, based on the number of people and fire control vehicles involved, the size of burned areas, and pre-fire track maintenance to limit the risks of fire escaping the compartment targeted for burning.

### Statistical analysis

We employed a suite of statistical approaches to determine whether Bitou Bush was a passenger or driver. These methods were used to quantify the effects of the components of the Bitou Bush treatment sequence on various metrics of native vegetation cover as well as their impacts on a suite of individual species of native plants. We also quantified the impacts of the occurrence of Bitou Bush on individual native plant species. In all models reported, we included site as a random term to allow for the likelihood that sites differed in their susceptibility to Bitou Bush infestation, and in the vegetation that would normally be found on those sites.

We fitted Hierarchical Generalized Linear Models (HGLM) [29] to our data on the number of individual Bitou Bush plants, percentage cover of live and dead Bitou Bush, native plant species richness, percentage cover of native plants, number of individual native plants, percentage crown cover, and the number of individuals of the most abundant species of native plants. For count data, we assumed a quasi-Poisson distribution and a gamma distribution with a log link function for the random component of the model [29]. Because observations of cover data appeared to be less variable for sites with very high or low cover, we used a quasi-binomial distribution with a logit link, and a beta distribution with a logit link for the random component (see [29]). We fitted random effects for both site and survey year to account for between-site differences not related to the treatments. Because many treatments were applied after surveys had commenced, comparison of treatment sequences is possible for individual sites, and estimates of treatment effects are based on the combination of both observed within-site differences and observed between-site differences. Thus, each treatment site acted as its own control given it was surveyed on a repeated basis after different sub-components of the treatment sequence.

We first fitted a model with disturbance sequence as the only predictor. We also constructed candidate predictors such as whether or not there had been a fire in the 12 months prior to a survey and whether or not there had been a fire in the 24 months prior to a survey. We also constructed similar predictors for spraying. Other predictors included the type of forest vegetation and the number of years from the commencement of the study.

Initially, we ignored the random structure and fitted all possible subsets of these predictors plus the sequence factor defined above. We ranked these models using the Schwarz Information Criterion (BIC) [30] and then fitted the best model for the fixed effects with year and site random effects included in the model. We used Wald tests to identify the terms that still had significant effects, and we dropped terms that were clearly non-significant at this stage. We investigated interactions between the remaining terms.

In an additional set of analyses, we quantified the effects of spraying and burning on a suite of individual species of native plants. We confined these analyses to plant species for which we recorded 400 or more individuals over the study duration. As part of these analyses, we also quantified the impacts of the occurrence of Bitou Bush *per se* on individual native plant species in the absence of spraying or fire.

We conducted all computations using either GenStat Release 16.1 or R version 3.0.2.

## Results

### Q1. Do control efforts result in a reduction in the cover of Bitou Bush and the abundance of live Bitou Bush plants?

**Fig 5:**
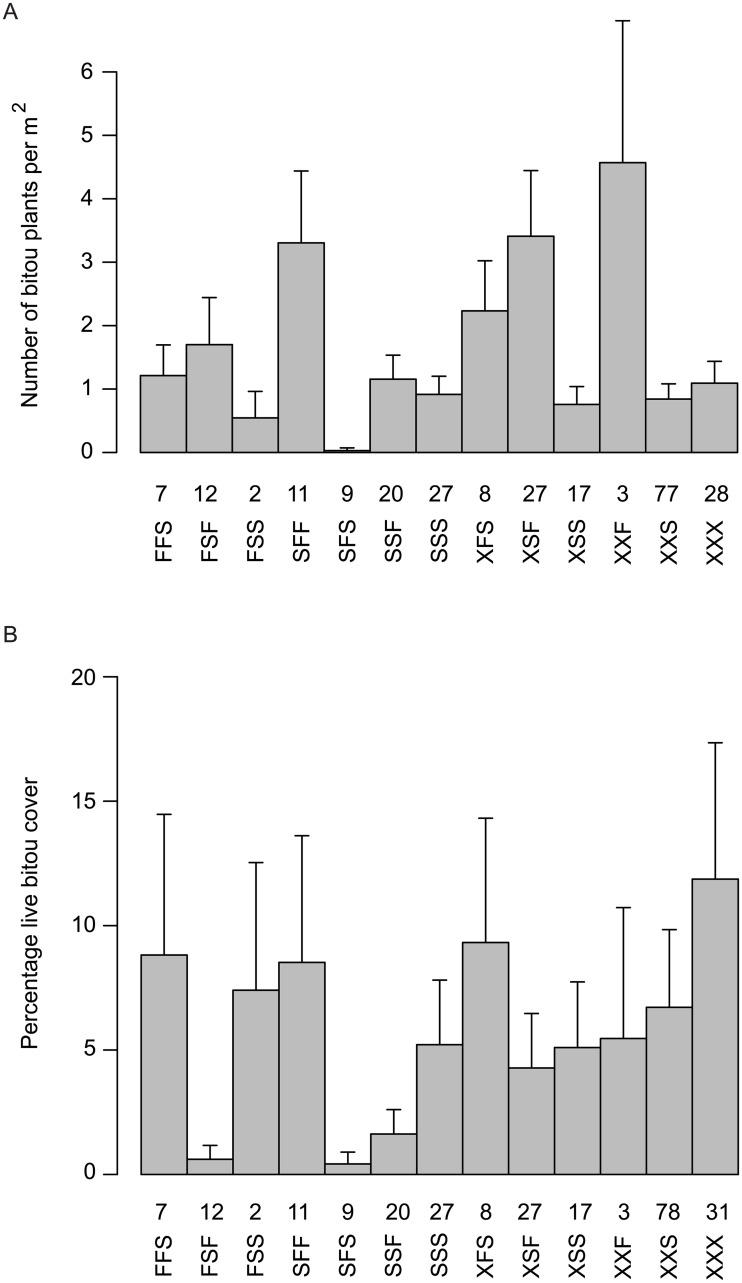
Treatment sequence effects on the number of individual Bitou Bush plants (A) and the amount of Bitou Bush cover (B). S denotes spray and F denotes fire. For example, the sequence spray-fire-spray is denoted SFS. Where there were fewer than three treatments an X denotes the missing event. The numbers above each treatment sequence correspond to the number of surveys completed for that sequence. Each column has an associated 95% confidence interval. A given site is surveyed many times throughout the duration of the study and can appear under different treatment sequences according to the progression of particular treatments over time (see text).

We observed highly significant treatment sequence effects ($\chi_{13}^{2}$ = 106.3, *P* < 0.001) on the abundance of live Bitou Bush plants. The treatment sequence of spray-fire-spray led to the lowest number of Bitou Bush plants (Fig 5a) and the lowest amount of live Bitou Bush cover (although similar to fire-spray-fire; see Fig 5b). Other treatment sequences reduced the abundance of live Bitou Bush plants, particularly fire-spray-spray (for number of plants) and fire-spray-fire (for live cover). We also identified highly significant year effects with the number of Bitou Bush plants decreasing by 27% per year ($\chi_{1}^{2}$ = 458.0, *P* < 0.001, 95% confidence interval: 21%-34%). Recent fire (within the previous two years) increased the number of Bitou Bush plants by 117% ($\chi_{1}^{2}$ = 37.5, *P* <0.001, 95% confidence interval: 68%-179%). Spraying within the previous two years reduced the number by33% ($\chi_{1}^{2}$ = 18.1, *P* <0.001, 95% confidence interval: 19%-45%).

**Fig 6:**
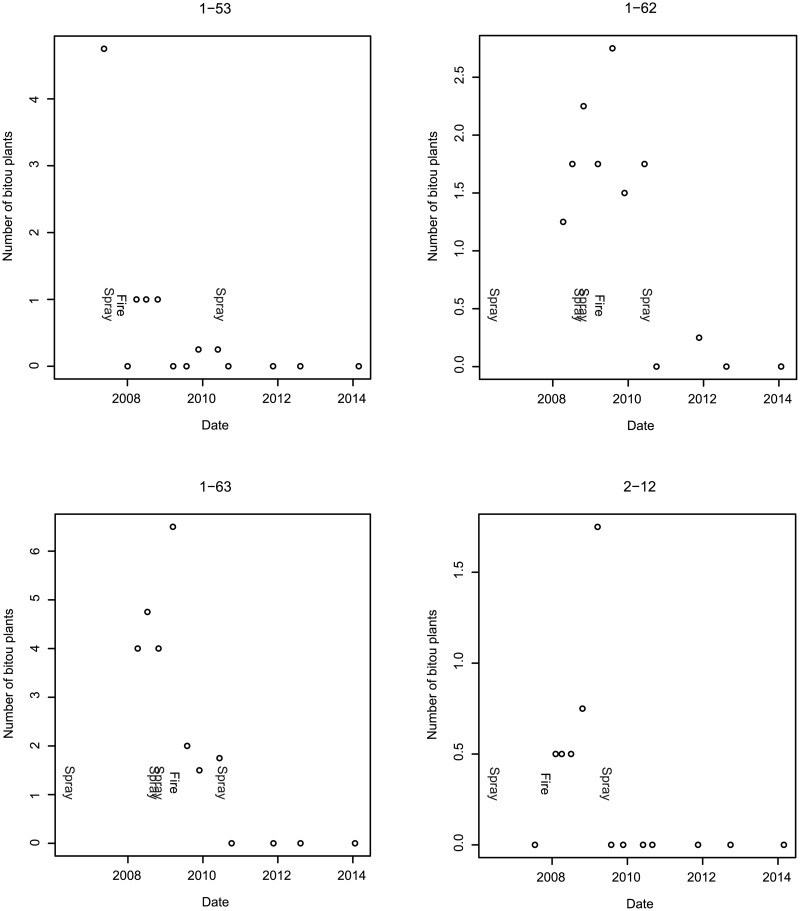
The numbers of individual Bitou Bush plants per m^2^ on four of the sites that received the treatment sequence spray-fire-spray. The codes at the top of each sub-figure correspond to a given site (e.g. 1–53).

**Fig 7:**
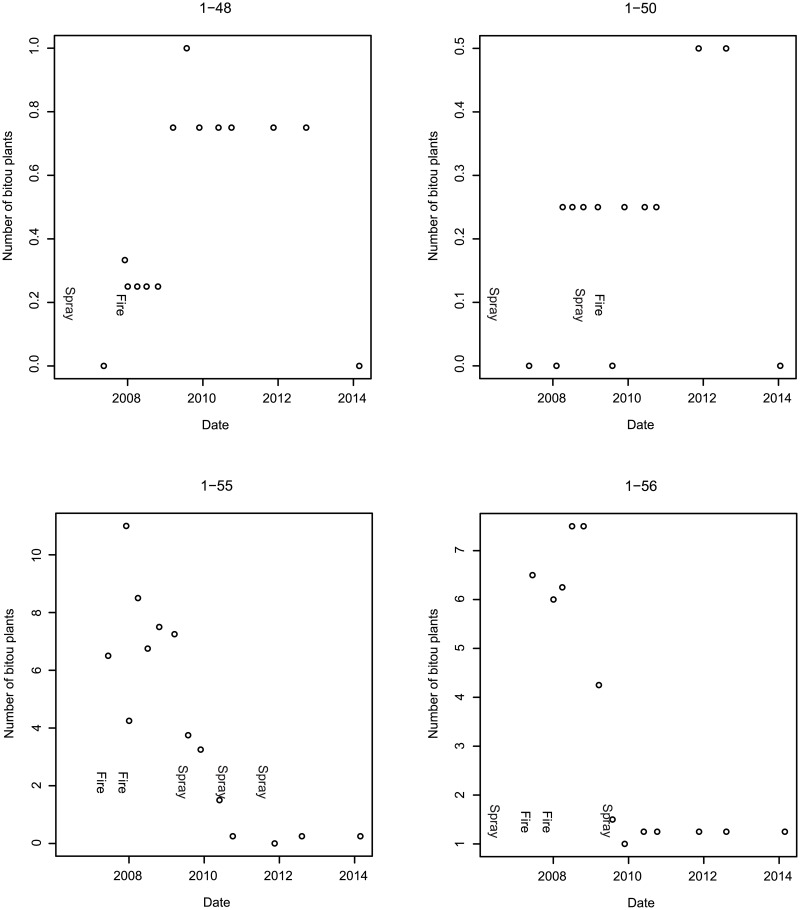
The numbers of individual Bitou Bush plants per m^2^ on four sites that received treatment sequences other than spray-fire-spray. The codes at the top of each sub-figure correspond to a given site (e.g. 1–48).

Fig 5 highlights the composite effects for sites in a given treatment sequence on the abundance of Bitou Bush (i.e. cover and number of plants). We decomposed these effects to examine site-specific responses for eight experimental sites (Figs 6 and 7). This underscored the importance of following fire with a spray treatment to achieve effective control. Our data showed that after spray-fire-spray, Bitou Bush abundance remained at zero or close to it for at least 2–3 years (Fig 6). Other treatment sequences such as spraying and fire (without subsequent spraying) led to a significant increase in the abundance of Bitou Bush (Fig 7).

### Q2: Do control efforts promote re-invasion by other exotic plant species?

After Bitou Bush, the next three most prevalent exotic plant species recorded in our surveys were rare. They were Creeping Woodsorrel *Oxalis corniculata* L. (89 individuals recorded across all sites in all years), Fleabane *Conyza bonariensis* (L.) (65 individuals recorded), and Black-berry Nightshade *Solanum nigrum* L. (17 individuals recorded). Bitou Bush removal did not lead to a significant increase in the abundance of these or any other (even rarer) exotic species (data not shown).

We found no evidence of re-invasion of Bitou Bush in treated sites 2–3 years after the spray-fire-spray treatment (Fig 6). Where only fire or spraying had been employed, there was evidence that incomplete treatment could increase the abundance of Bitou Bush in the following 1–2 years (Fig 7).

### Q3: What are the effects of the Bitou Bush control program on native plants?

**Fig 8:**
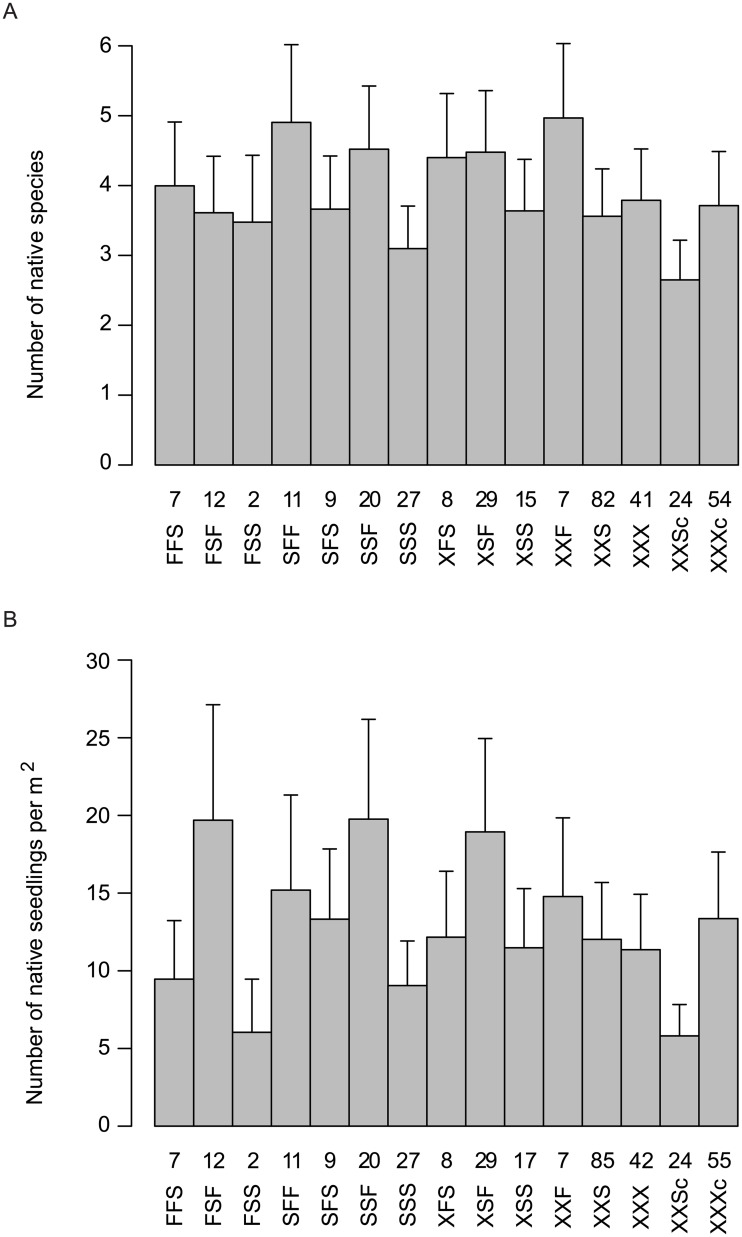
Effect of treatment sequences used in the control of Bitou Bush on number of native plant species (A) and number of native seedlings (B). The sequence spray-fire-spray is denoted SFS. Where there were fewer than three treatments an X denotes the missing event. The numbers above each treatment sequence correspond to the number of surveys completed for that sequence. Each column has an associated 95% confidence interval.

Treatment sequences had highly significant effects on the numbers of native plant species ($\chi_{15}^{2}$ = 94.8, *P* < 0.001) and native plant seedlings ($\chi_{15}^{2}$ = 118.0, *P* < 0.001; Fig 8). These results include sites where there was no spraying or fire.

We also found a significant treatment sequence effect ($\chi_{13}^{2}$ = 59.2, *P* < 0.001) on Bracken Fern, *Pteridium esculentum* (G.Forst.) Cockayne, with sequences encompassing both spraying and fire leading to a peak in the number of emergent stems. Table 2 shows the response of native vegetation to recent spraying and fire over time. Spraying in the two years prior to a survey reduced the number of native plant species and the number of native seedlings present.

**Table 2:** Responses of native vegetation to recent spraying, recent fire and time, as estimated percentage change with a 95% confidence interval.

	Spraying in previous two years		Fire in previous two years		Annual change	
Response	% Change	*P*	% Change	*P*	% Change	*P*
Number of native species	-14% (-20%- -8%)	<0.001	17% (7%- 29%)	<0.001	20% (5%- 37%)	0.006
Number of native seedlings	-31% (-41%—-20%)	<0.001	44% (21%- 72%)	<0.001	35% (3%- 77%)	0.026
Number of grass species	-4% (-10%- 3%)	0.28	-11% (-19%- 3%)	0.010	26% (7%- 48%)	0.004
Number of bracken stems	-7% (-21%- 9%)	0.35	57% (29%- 92%)	<0.001	4% (-7%- 16%)	0.48

A negative percentage change indicates a reduction in a response; a positive percentage change indicates an increase in a response.

Nine of the 19 individual species we examined exhibited a significant (P < 0.05) negative response to spraying with the level of change ranging from 18% to 67% reduction (Table 3). Seven of the 19 individual plant species were significantly (P < 0.05) less likely to occur in the presence of Bitou Bush (Table 3). That is, their estimated abundance was negatively related to the abundance of Bitou Bush (Table 3). The mixed responses to fire observed among different plant metrics was mirrored in our analyses of individual plant species. Some, such as the herb *Glycine tabacina* (Labill.) Benth. and Snake Vine *Stephania japonica* (Thunb.) Miers, exhibited a marked increase following fire. Others (e.g. the grasses *Microlaena stipoides* (Labill.) R.Br. and *Entolasia marginata* (R.Br.) Hughes) declined markedly after fire (Table 3).

**Table 3:** Estimated numbers of individual native plants per 100 m^2^, and percentage change in the numbers of plants after fire and/or spray in the 1–2 years preceding a survey.

Species	Life form	Plants per 100 m^2 (a)^	Fire	Spray	*C*. *monilifera* cover	Time
*Acianthus fornicatus*	Herb	72.8		-37%	-17%	
*Commelina cyanea*	Herb	44.4	-38%	-31%		
*Cyperus gracilis*	Sedge	49.6	50%	-24%	15%	75%
*Desmodium varians*	Herb	92.5				32%
*Dichondra repens*	Herb	78.4			-21%	77%
*Entolasia marginata*	Grass	95.0	-46%		30%	
*Geranium homeanum*	Herb	52.2		-46%		70%
*Glycine tabacina*	Herb	37.8	48%			58%
*Imperata cylindrica*	Grass	399.6	-28%	-18%	-12%	15%
*Lomandra longifolia*	Herb	78.6	52%			22%
*Microlaena stipoides*	Grass	237.5	-50%	-33%		61%
*Oplismenus aemulus*	Grass	261.2		-48%		352%
*Oplismenus imbecillis*	Grass	44.1	-60%			
*Parsonsia straminea*	Vine	111.5	125%			195%
*Poranthera microphylla*	Herb	55.4				
*Pteridium esculentum*	Fern	152.2	77%		-15%	
*Schelhammera undulata*	Herb	91.7	-36%		-38%	
*Stephania japonica*	Vine	45.9	29%	-36%	-17%	
*Viola hederacea*	Herb	70.7	206%	-67%	-42%	41%

We also present the estimated percentage change in the abundance of individual plant species response in to an increase in Bitou Bush cover of a tenth of the area, and the percentage change per year in the absence of spray, fire or change in the cover of Bitou Bush. Only effects significant at the 5% level are shown. Information on life forms are based on data in PlantNET [31]. We present data for the 19 individual species for which 400 or more individuals were recorded.

^**a**^Based on data from 1 m x 1 m survey plots (see text).

### Q4: What is the cost-effectiveness of Bitou Bush control?

We calculated the costs (on a ha^-1^ basis) of the different treatment sequences and compared them with their effects on the abundance of both Bitou Bush and native plant species. This enabled comparison of relative cost-effectiveness of different sequences of sub-treatments (Table 4). Spray-fire-spray ranked best among treatment sequences in terms of Bitou Bush control, close to the top in terms of native plant species richness and cover, but was relatively expensive compared to some other treatment sequences (Table 4, Fig 8). Sequences like fire-fire-spray were more costly than spray-fire-spray (Table 4) but somewhat less effective in controlling Bitou Bush (Fig 5). Less expensive options such as a single spray event had limited controlling effects on Bitou Bush (Fig 5), but significant negative effects on native plant species (Tables 2 and 3, Fig 8). Comparatively costly sequences like two spraying events followed by fire ranked moderately well in terms of Bitou Bush control and best for species richness. Fire without spraying exacerbated the invasive plant species problem by leading to an increase in Bitou Bush abundance and cover (Fig 5). Yet, other treatment sequences such as fire followed by a series of three spraying events resulted in a significant reduction in the number of Bitou Bush plants (Fig 5). However, in this case, there were no significant differences the number of Bitou Bush plants after one spraying event compared with three post-fire spraying events (Fig 5), suggesting no additional benefits were derived from the added costs of extra spraying.

**Table 4:** Summary of the estimated ha^-1^ costs (in 2012 Australian dollars) of treatment sequences for Bitou Bush, the effectiveness of weed control and impacts on native plant species and cover (as a percentage of the results where no treatment had been applied in the previous four years (denoted by XXX)).

Sequence	Cost ha^-1^($A)	Number of *C*. *monilifera*	% Cover Live *C*. *monilifera*	Number of native species	% Native Cover
SFS	1440	0.03 (1)	0.4% (1)	97% (6)	110% (5)
FSS	1440	0.54 (2)	7.4% (9)	92% (2)	115% (11)
XSS	290	0.76 (3)	5.1% (5)	96% (5)	112% (8)
XXS	145	0.84 (4)	6.7% (8)	94% (3)	112% (9)
SSS	435	0.92 (5)	5.2% (6)	82% (1)	111% (7)
XXX	0	1.09 (6)	11.9% (13)	100% (7)	100% (2)
SSF	1440	1.16 (7)	1.6% (3)	119% (11)	103% (3)
FFS	2445	1.21 (8)	8.8% (11)	105% (8)	110% (6)
FSF	2445	1.7 (9)	0.6% (2)	95% (4)	96% (1)
XFS	1295	2.23 (10)	9.3% (12)	116% (9)	104% (4)
SFF	2445	3.3 (11)	8.5% (10)	129% (12)	122% (12)
XSF	1295	3.41 (12)	4.3% (4)	118% (10)	135% (13)
XXF	1150	4.57 (13)	5.5% (7)	131% (13)	114% (10)

The sequences are listed in order of their effects on the mean number of live Bitou Bush plants per survey plot. The numbers in parentheses in columns 3–6 are the relative ranking in terms of effects; 1 is best for Bitou Bush control whereas 13 is best in terms of native vegetation cover and number of native species. The impact on native plants is given as a percentage of the value for the disturbance-free sites.

## Discussion

Published information is often lacking on the effectiveness of interventions in natural resource management, including programs designed to eradicate or control invasive plant species [32]. Yet it is critical to know the effectiveness of such actions to make well targeted, successful and cost-effective interventions [11,23]. This is particularly important in invasive species management because the relative success of control can vary according to many factors such as the stage of invasion that a given species is in, the supply of invasive and native species propagules from the soil seedbank and surrounding populations [26], the extent of non-target impacts on native communities, and the capacity of native flora to colonise and regenerate [6,33]. To explore some of these key issues, we took advantage of a major, applied invasive species management program in an iconic National Park in south-eastern Australia to complete a detailed, 7-year experimental case study of Bitou Bush control. Four key findings of our comparative experimental study include:

1. Bitou Bush was a driver of environmental change. We reached this conclusion for two reasons. First, we found no evidence that the spray-fire-spray treatment regime increased the susceptibility of treated areas to re-invasion by Bitou Bush or by other exotic plant species within 2–3 years after the last spray. Second, native plant species richness and cover increased over time (Table 3) and approached levels characteristic of control sites where Bitou Bush was absent (Table 2). Thus, our findings suggest that removal of Bitou Bush could lead to native plant recovery, indicating ecosystem release from one, apparent (exotic) driver of change. Notably, the recovery of native plant species in our experiment suggests that seed limitation of native species is no greater in areas formerly invaded by Bitou Bush and those where Bitou Bush has been absent. It is possible that Bitou Bush may have responded to other drivers of change in our two target vegetation types (such as altered fire regimes), but we examined for (and found little evidence of) the effects of them using control plots in the experimental design.
2. The full treatment (spray-fire-spray) protocol significantly reduced Bitou Bush. The number of Bitou Bush plants then remained at, or close to, zero for at least 2–3 years after the final spray event (Fig 6). This result, coupled with the limited and relatively short-lived impacts of this treatment sequence on native vegetation cover, suggested that spray-fire-spray was the most cost-effective form of Bitou Bush control.
3. Outcomes of weed control were often strongly dependent on the treatment sequence deployed. Some incomplete treatment sequences appeared to be relatively ineffective or even increased the abundance of Bitou Bush (Fig 8).
4. In the event of an (unplanned) wildfire in an area infested with Bitou Bush, our results suggest it can be useful to follow such natural disturbance with spraying (at the appropriate time of the year) to kill the pulse of seedlings likely to germinate after the fire.

### Q1: What are the direct effects of the control method on Bitou Bush and is there an effect of the treatment sequence?

Our study suggested that spray-fire-spray was the best treatment sequence for reducing the number and cover of live Bitou Bush plants (Figs 5 and 6). This apparent effectiveness is consistent with the known biology of the species in which seeds in the soil are triggered to germinate following fire but these germinants are then killed by follow-up spraying [13,14]. Conversely, evidence suggests that fire without spraying led to an increase in the abundance of Bitou Bush, highlighting the importance of control methods (and the sequence of treatments within the protocol) that are tightly aligned to the ecology of the target plant species. We readily acknowledge that despite the comparative success of the spray-fire-spray as the best treatment sequence, constant monitoring, evaluation and potentially follow-up control will be required, especially as birds and other animals may continue to bring Bitou Bush seeds into the study area.

### Q2. Do control efforts promote re-invasion by Bitou Bush or other exotic plant species?

The so-called “success” of a weed control program needs to be gauged relative to objectives [18]. Perverse outcomes can arise if the sole measure of success is a reduction of the target invasive species without consideration of the potential for re-invasion by that species or other non-native plants (Fig 1) (e.g. [4,6,17]). However, we found no evidence of a significant level of re-invasion of Bitou Bush treated sites where the spray-fire-spray treatment had been applied for at least three years after the final spray (Fig 7). Our study has continued for seven years but as outlined above and given the potential risks of re-invasion, ongoing surveys of vegetation cover (including those of the prevalence of Bitou Bush) are critical. This will help determine how frequently control efforts need to be employed.

Other investigations (e.g. see [17]) have found that efforts to remove Bitou Bush led to colonization by other invasive plants. Reasons for differences between earlier work and this investigation remain unclear, but may be associated with differences in site histories (e.g. presence or absence of sand mining), herbivore densities and grazing pressure [34], differences in the suite of other invasive species in the study areas [17], or the possibility that Bitou Bush may act as a transformer in other ecosystems (sensu [8]) such that only exotic species can recolonise and native plant taxa are excluded. In addition, the area where we conducted this detailed case study is somewhat isolated from other locations in which Bitou Bush infestation is a major problem. Such differences (e.g. in the composition of propagule pools) may have contributed to the differences between our results and those of other studies (e.g. [17]).

### Q3. What are the effects of the Bitou Bush control program on native plants?

Our results suggest that Bitou Bush was a driver and not a passenger or transformer of environmental change (see Fig 1). As a result, when appropriate treatment is employed, this invasive species can be effectively removed, re-invasion is limited, and native plant species can begin to recover. We based this conclusion on strong relationships between spraying and burning treatments and native plant species richness, native plant cover, and individual plant species abundance (Fig 8, Table 3). Our findings were broadly consistent with several earlier studies [6,7,18,21,35,36], although as outlined above, other workers have found that the removal of Bitou Bush can be followed by the establishment of other exotic plant species [17].

Fire typically led to an increase in most measures of native plant species richness and/or cover, whereas spraying had the opposite effect. Measures like the number of native plant seedlings and native grasses exhibited a significant positive increase over time, suggesting the negative effects of spraying were relatively short-lived (1–2 years). Our results also suggest that the increases in native plant species richness and cover over time occurred whilst there was a concurrent decline in the number of live Bitou Bush plants (Table 3). We therefore conclude that the removal of the target invasive plant species is providing opportunities for native vegetation recovery, although some individual species of native plants were significantly negatively affected by spraying or burning (Table 3). Indeed, we found a strong negative response to spraying with the level of change ranging from a 19% to 60% reduction (Table 3). These results are consistent with those of other researchers (see [37]) but appear to be counter to the current perception that ULV glyphosate used in winter for Bitou Bush control has only limited impact on native plant species. Moreover, two spray treatments further reduced the number of native plant species. There is therefore a need for careful application of herbicides coupled with ongoing monitoring to determine native plant recovery trends over time.

Our results suggesting that Bitou Bush was a driver rather than a passenger or transformer of change meant that is was not necessary to explicitly address these questions in our decision tree (i.e. scenarios 3–6 in Fig 1) or to consider whether a novel plant community had developed following Bitou Bush control. However, there may be situations where this is not the case [38].

### Q4. What is the cost-effectiveness of Bitou Bush control?

Few invasive plant control programs attempt to integrate data on the ecological effectiveness and the cost-effectiveness of a weed control program [12] (but see[11] as well as a number of studies of invasive plants in agricultural crops). Yet, integration of costs in management assessment is important because only limited funds are available for weed control. We suggest the point at which cost becomes important is when there is more than one possible control action that meets the management objective (see Fig 1). This was not the case for Bitou Bush. That is, we could not identify an alternative treatment sequence to spray-fire-spray which had the same controlling effect on Bitou Bush while at the same time minimizing the negative impacts on native vegetation.

Of the treatment sequences we examined, the current spray-fire-spray protocol had the greatest controlling effect (Fig 5). Other treatment sequences were cheaper, but often exhibited less effective control on Bitou Bush (Table 4). Some alternative treatment sequences led to perverse outcomes through increasing Bitou Bush cover and/or abundance (Fig 7). In terms of cost-benefit assessment, these treatments were not cost-effective, and only the full spray-fire-spray sequence yielded cost effectiveness in terms of outcomes. This has important implications, given resourcing issues associated with implementing the full treatment sequence. However, there can sometimes be opportunities to improve cost-effectiveness such as after unplanned fires in areas infested with Bitou Bush to spray the pulse of germinants after fire. This would be particularly cost-effective as fire management is the most expensive component of the treatment sequence protocol. Hence, managers should be alert to the potential ecological and financial benefits that can arise from unplanned events. Application of post-fire spraying would need to be timed appropriately to limit its impacts on native plant species. Moreover, as many weed control programs have sub-components of a full treatment protocol and each step has a monetary cost, there may be opportunities for savings by eliminating unnecessary, non-significant sub-components. As an example from this study, repeated spraying events following wildfire were unnecessary as the number of individual Bitou Bush plants was reduced close to zero after the first round of post-fire spraying (Fig 5).

## Conclusion

We explored the efficacy of efforts to control a highly invasive plant species and to facilitate the restoration of native plant species richness to levels characteristic of uninvaded sites. When an appropriate sequence of sub-treatments is employed, Bitou Bush can be removed, re-invasion is limited, and native plant species can begin to recover. Critical to success is avoiding treatment sequences that may exacerbate invasive plant species problems. However, we also showed there can be value in taking advantage of unplanned events such as wildfires to deploy follow-up spraying to kill the pulse of seedlings likely to germinate after the fire (e.g. [39]).
